# Predictors of Mortality in Patients With Dengue Fever: Insights From a Comparative Analysis

**DOI:** 10.7759/cureus.36040

**Published:** 2023-03-12

**Authors:** Afsheen Mahmood, Anwar ul Haq, Said Amin, Fawad Rahim, Mohammad Noor, Huma Gul, Sheraz Zafar, Sobia Ahmed Qureshi, Khaula Batul, Mohsina Haq

**Affiliations:** 1 Internal Medicine, Hayatabad Medical Complex, Peshawar, PAK; 2 Internal Medicine, Khyber Girls Medical College, Peshawar, PAK; 3 Internal Medicine, Hayatabad Medical Complex Peshawar, Peshawar, PAK; 4 Microbiology, Peshawar Medical College, Peshawar, PAK

**Keywords:** risk factor, determinant, acute kidney injury, alanine aminotransferase, c reactive protein, thrombocytopenia, leukocytosis, fatal outcome, mortality, dengue fever

## Abstract

Objective

To determine the clinical and biochemical predictors of mortality in patients with dengue fever.

Methods

This was an analytical, cross-sectional study conducted at Hayatabad Medical Complex, Peshawar, Pakistan. The study participants were patients admitted to the hospital for the management of dengue fever. Clinical parameters (age, gender, duration of hospital stay, and the presence of complications) and biochemical parameters [white blood cells count (WBC), platelet count, serum c-reactive protein (CRP) level, serum alanine aminotransferase (ALT) level, and serum creatinine] were recorded. These parameters were compared between the survivors and non-survivors of dengue fever.

Results

Out of 115 patients, the majority (n=82, 71.3%) were up to 45 years and the mean age was 38.40 ± 18.1 years. Most of the patients (n=105, 91.3%) survived. On univariate logistic regression analysis, age more than 45 years [odds ratio (OR) 0.141, 95% confidence interval (CI) 0.034 - 0.585, p = 0.007), leukocytosis (> 11,000/mcL) (OR 0.187, 95% CI 0.049 - 0.719, p = 0.015), and acute kidney injury (creatinine > 1.5 mg/dL) (OR 0.124, 95% CI 0.029 - 0.531, p = 0.005)] at the time of admission reduced the likelihood to survive. Leukocytosis and acute kidney injury remained significant independent predictors of mortality on multivariate logistic regression analysis. [(OR 0.201, 95% CI 0.042 - 0.960, p = 0.044) and (OR 0.148, 95% CI 0.026 - 0.857, p = 0.033) for survival, respectively]. Gender, duration of inpatient stay, thrombocytopenia (platelets < 30,000/mcL), and acute liver injury (ALT > 200 IU/L) were not associated with mortality from dengue fever.

Conclusion

Age over 45 years, leukocytosis, and acute kidney injury at presentation increased the likelihood of mortality from dengue fever in this study. Gender, duration of hospital stay, thrombocytopenia, and acute liver injury did not affect the odds of mortality.

## Introduction

Benjamin Rush was the first to describe dengue fever in the late 18th century [1]. Dengue is an arboviral illness, and female Aedes aegypti (A. aegypti) and Aedes albopictus (A. albopictus) are the main vectors. It is a single-strand ribonucleic acid (RNA) virus and has four serotypes (DEN1 to DEN4) [2]. Dengue is a disease of tropical and subtropical areas with frequent epidemics during the rainy season [3]. However, it is a major public health problem due to increased travel. It is an important cause of acute febrile illness in people living in and traveling from the endemic regions.

The symptoms of dengue fever include asymptomatic seroconversion to classic acute febrile illness with severe body aches, dengue hemorrhagic fever, and dengue shock syndrome. Abdominal pain is more common in patients who have had a dengue virus infection before. Hematological and biochemical abnormalities include cytopenias, deranged liver function tests, and elevated inflammatory markers like serum c-reactive protein (CRP) and serum ferritin. The diagnosis of acute dengue fever is confirmed by polymerase chain reaction (PCR) or by demonstrating dengue non-structural antigen-1 (NS-1) and/or dengue-specific IgM [4].

The first case report of dengue fever in Pakistan dates back to 1982. Since the beginning of this century, there has been an epidemic almost every year with prolonged duration, a higher number of cases, and increasing severity. The reasons for this changing epidemiology include climate change, frequent natural disasters like flooding and earthquakes, and the war on terror leading to internally displaced people, particularly in Khyber Pakhtunkhwa province of Pakistan. Moreover, the resistance of the vector to insecticides and changing virulence of the virus serotypes also contribute to the changing epidemiology [5].

At the moment, there is no approved vaccine or medication for the dengue virus. To reduce the incidence and mortality from dengue fever, the emphasis should be on vector control, community awareness, proper training of the health care personnel, triage of cases at higher risk for complications, and timely treatment as per validated protocols.

Research has been conducted to determine the clinical and laboratory parameters which can predict patients at higher risk of complications and mortality. The laboratory parameters include full blood count (FBC), arterial blood gases (ABG), and serum lactate level [6,7]. Gall bladder wall edema has been reported as an early feature of dengue leak syndrome [8]. The results of these parameters are conflicting. We aim to determine the clinical and biochemical parameters predictive of mortality in dengue fever in patients admitted to a teaching hospital.

## Materials and methods

This analytical, cross-sectional study was performed in the Department of Internal Medicine, Hayatabad Medical Complex, Peshawar, Pakistan after approval by the institutional review board of Khyber Girls Medical College, Peshawar, Pakistan. All patients admitted for the management of dengue fever between September 8th to November 18th, 2022, were eligible for the study. Patients who consented to take part were enrolled in the study.

All patients underwent history taking and physical examination, and basic laboratory investigations were requested from the hospital laboratory for all the patients. The following parameters were recorded for every patient at the time of admission: age, gender, white blood cells count (WBC), platelet count, serum c-reactive protein (CRP) level, serum alanine aminotransferase (ALT) level, and serum creatinine. The duration of stay in the hospital and the presence of any complication of dengue fever was documented by following all the patients.

The outcome for each patient was recorded as survivors for those who recovered and were discharged from the hospital or non-survivors for those who died during the hospital stay. Thrombocytopenia was defined as a platelet count of less than 30,000/mcL, leukocytosis as WBC of more than 11,000/mcL, elevated CRP as a level of more than 5 mg/dL, acute liver injury as ALT level above 200 IU/L, and acute kidney injury as serum creatinine above 1.5 mg/dL.

Data were analyzed with SPSS (IBM Corp. Released 2012. IBM SPSS Statistics for Windows, Version 21.0. Armonk, NY: IBM Corp). Means and standard deviations were determined for age and duration of hospital stay. Frequencies and percentages were determined for categorical variables. Differences in the means of age and duration of hospital stay of survivors and non-survivors were evaluated for statistical significance using the Mann-Whitney U test. Similarly, the categorical variables were evaluated using the chi-square test/Fisher’s exact test. Statistically significant variables like age groups, leukocytosis, and acute kidney injury were analyzed using univariate and multivariate logistic regression analysis to eliminate the effect of confounders. A p-value of less than or equal to 0.05 was taken as significant. Results are shown in the form of tables. 

## Results

A total of 115 patients were enrolled in the study. The mean age of the study participants was 38.40 ± 18.1 years. Overall, most of the patients were up to the age of 45 years (n=82, 71.3%) and male (n=73, 63.5%). There were no complications in 57.4% (n=66) patients and 105 (91.3%) patients survived the disease. Details of the study subjects are presented in Table 1.

**Table 1:** Characteristics of study subjects (n=115) SD: Standard deviation

Age, Mean ± SD (years)	38.40 ± 18.1
Age groups, No. (%)	
Up to 45 years	82 (71.3%)
More than 45 years	33 (28.7%)
Gender, No. (%)	
Male	73 (63.5%)
Female	42 (36.5%)
Duration of hospital stay, Mean ± SD (days)	4.6 ± 3.4
Complications*, No. (%)	
No complications	66 (57.4%)
Acute liver injury	13 (11.3%)
Acute kidney injury	12 (10.4%)
Gastrointestinal hemorrhage	10 (8.7%)
Shock	08 (7%)
Encephalitis / Meningitis	05 (4.3%)
DIC	04 (3.5%)
Cholecystitis	04 (3.5%)
Outcome, No. (%)	
Survivors	105 (91.3%)
Non-survivors	10 (8.7%)

Compared to the non-survivors, the survivors were significantly younger (51.2 ± 18.1 vs 38.4 ± 18.1 years, p = 0.024). A statistically significant number of non-survivors were over the age of 45 years (70% vs 24.8%, p = 0.006). The distribution of males and females among the survivors and non-survivors did not differ significantly (p = 0.275). Non-survivors had a longer hospital stay than the survivors (7.5 ± 9.7 vs 4.3 ± 2.0 days) but the difference did not achieve statistical significance (p = 0.326). Compared to survivors, a significantly higher number of non-survivors had leukocytosis (60% vs 21.9%, p = 0.016), elevated CRP (100% vs 61%, p = 0.010), and acute kidney injury (40% vs 7.6%, p = 0.010) at the time of admission. Thrombocytopenia and acute liver injury at arrival was not associated with mortality (Table 2).

**Table 2:** Comparison of clinical and laboratory parameters between survivors and non-survivors *P value from Fisher’s exact test SD: Standard deviation, mcL: microliter, mg/dL: milligram per deciliter, ALT: Alanine aminotransferase, U/L: Unit per liter

Variables	Outcome		p-value
	Non- Survivors (n=10)	Survivors (n=105)	
Age, Mean ± SD (years)	51.2 ± 18.1	38.4 ± 17.7	0.024
Age groups, No. (%)			
Up to 45 years	03 (30%)	79 (75.2%)	0.006*
More than 45 years	07 (70%)	26 (24.8%)	
Gender, No. (%)			
Male	05 (50%)	68 (64.8%)	0.275*
Female	05 (50%)	37 (35.2%)	
Duration of hospital stay, Mean ± SD (days)	7.5 ± 9.7	4.3 ± 2.0	0.326
Laboratory parameters at admission, No. (%)			
Thrombocytopenia (< 30,000/mcL)			
Yes	02 (20%)	32 (30.5%)	0.386*
No	08 (80%)	73 (69.5%)	
Leukocytosis (> 11,000/mcL)			
Yes	06 (60%)	23 (21.9%)	0.016*
No	04 (40%)	82 (78.1%)	
Elevated c-reactive protein (> 5 mg/dL)			
Yes	10 (100%)	64 (61%)	0.010*
No	00 (0%)	41 (39%)	
Acute liver injury (ALT > 200 U/L)			
Yes	01 (10%)	12 (11.4%)	0.685*
No	09 (90%)	93 (88.6%)	
Acute Kidney Injury (S. creatinine > 1.5 mg/dl)			
Yes	04 (40%)	08 (7.6%)	0.010*
No	06 (60%)	97 (92.4%)	

Logistic regression analysis revealed that compared to patients younger than 45 years, older patients had a significantly lower likelihood of survival [unadjusted odds ration (OR) 0.141, 95% confidence interval (CI) 0.034 - 0.585, p=0.007)]. The odds of survival for the older patients were not significant when adjusted for the presence of leukocytosis and acute kidney injury in multivariate logistic regression analysis (adjusted OR 0.309, 95% CI 0.063 - 1.512, p=0.147). Patients with leukocytosis at admission were less likely to survive (unadjusted OR 0.187, 95% CI 0.049 - 0.719, p=0.015; adjusted OR 0.201, 95% CI 0.042 - 0.960, p=0.044). Similarly, patients with acute kidney injury (AKI) at the time of admission were significantly less likely to survive than those without AKI (unadjusted OR 0.124, 95% CI 0.029 - 0.531, p=0.005; adjusted OR 0.148, 95% CI 0.026 - 0.857, p=0.033) (Table 3).

**Table 3:** Logistic regression analysis for predictors of survival OR: Odds ratio, CI: Confidence interval

Variables		Univariate analysis		Multivariate analysis	
		OR (95% CI)	P value	OR (95% CI)	P value
Age group	< 45 years				
	> 45 years	0.141 (0.034 – 0.585)	0.007	0.309 (0.063 - 1.512)	0.147
Leukocytosis	No				
	Yes	0.187 (0.049 – 0.719)	0.015	0.201 (0.042 – 0.960)	0.044
Acute kidney injury	No				
	Yes	0.124 (0.029 – 0.531)	0.005	0.148 (0.026 – 0.857)	0.033

## Discussion

Dengue fever outbreaks are common in the tropical and subtropical zones of the world. Its epidemiology is changing and its incidence has increased considerably over the past few decades [9]. Most symptomatic patients have a mild disease but complications leading to death do occur. It is one of the common causes of acute febrile illness in the monsoon season. Identifying clinical and laboratory parameters that may identify patients at risk of the worst outcome have been an area of active research. Most of the literature has focused on laboratory parameters that predict the severity of dengue fever rather than mortality. There are conflicting observations regarding the determinants of poor outcomes in dengue fever in regional and global research. This study is an endeavor to identify the determinants of mortality of dengue fever patients admitted to a teaching hospital in Peshawar, Pakistan.

The case fatality rate was 8.7%. It has ranged from 1.1% to 14% in studies from different regions of the world [10,11]. The variability might be due to the higher number of patients with complications at presentation in some studies, and the quality of treatment they received. Patients who received intensive care had a mortality rate of up to 21% [12].

Age plays a decisive role in determining the outcome of acute and chronic diseases. Patients who survived till discharge were younger compared to those who succumbed to the disease. A significant number of non-survivors were over the age of 45 years. Age more than 45 years was a significant predictor of mortality on univariate analysis but insignificant when adjusted for other variables. Karunakaran et al. and Jain et al. from India, and Mallhi et al. from Malaysia have reported increased mortality with increasing age [10,13,14]. In contrast, Bhaskar et al. from India and Khalil et al. from Pakistan did not find age as a predictor of mortality among patients with dengue fever [11,15]. The mortality rates did not differ significantly between male and female patients. This is in harmony with the findings of Bhaskar et al. from India [11].

Non-survivors stayed longer in the hospital than survivors, however, the difference did not achieve statistical significance. Khalil et al. from Karachi, Pakistan reported that increased length of hospital stay was associated with higher mortality [15]. This might be due to the fact that patients who develop complications of dengue fever were likely to stay longer and, given the severity of the disease, they had relatively higher mortality.

Leukopenia is a characteristic finding in patients with dengue fever, and it has been linked to the severity of the illness [16]. In contrast, a significantly higher number of non-survivors than survivors had leukocytosis. Leukocytosis predicted higher mortality on both univariate and multivariate logistic regression. This is similar to the observations of Thein et al. from Singapore and Almas et al. from Pakistan where non-survivors had a significantly higher WBC at the time of presentation [6,17]. Leukocytosis in patients with dengue fever should alert the attending physician to anticipate impending complications.

Similarly, compared to survivors, a significant number of non-survivors had elevated CRP at admission. Medagama et al. from Sri Lanka have comparable findings where elevated CRP increased the likelihood of death from dengue fever. C-reactive protein is an acute-phase protein, and it has been suggested that it may be a useful biomarker for predicting severe disease and poor outcomes [18].

Although acute kidney injury is not a common feature of dengue fever. However, AKI at the time of presentation increased the odds of mortality. Post hoc analysis showed that AKI is an independent factor of mortality in dengue fever. Acute kidney injury has been a consistent contributing factor to mortality in dengue fever [6,10-12,15].

Thrombocytopenia is a consistent laboratory finding of dengue fever. Bhsakar et al. from India have reported increased mortality among patients with thrombocytopenia (< 20,000/mcL) at presentation [11]. In contrast, thrombocytopenia at presentation was not associated with mortality in this study. Similar observations have been stated by Thein et al., Karunakaran et al., and Khalil et al [13,15,17].

Researchers have been divided on the role of ALT in predicting the outcome of cases with dengue fever. We observed that acute liver injury at admission did not affect the outcome. This is similar to the observations of Thein et al. and Karunakaran et al., but in contrast with those reported by Almas et al., Huang et al., Padyana et al., and Medagama et al. where hepatitis was reported to increase the odds of death from dengue fever [6,12,13,17-19].

The study addresses an important issue of identifying determinants of mortality in patients with dengue fever which can inform clinical decision-making and improve patient outcomes. Multivariate logistic regression analysis to adjust for the confounding factors adds to the robustness of the findings. The single-center setting of the study has a relatively small sample of only inpatients thus limits its generalization.

## Conclusions

This study determined the predictive factors for mortality in patients with dengue fever admitted to a teaching hospital. The case fatality rate was 8.7%. Age over 45 years, leukocytosis, and acute kidney injury at presentation were found to be significant predictors of mortality in this study. Gender, length of hospital stay, thrombocytopenia, and ALT levels were not found to be significant predictors of mortality. There is a need for further research to identify other potential risk factors and to establish a more definitive set of predictors for mortality in patients with dengue fever.

## References

[1] Barnett R (2017). Dengue. Lancet.

[2] Shah MM, Shah N, Iftikhar M, Abbas S, Rahim F, Jamal A (2021). Frequency of pruritic rash in patients with dengue fever presenting to a tertiary care hospital. Khy J Med Sci.

[3] Jahan F (2011). Dengue Fever (DF) in Pakistan. Asia Pac Fam Med.

[4] Rahim F, Amin S, Noor M, Ali B, Wahab A (2022). Dengue fever, Crimean-Congo hemorrhagic fever, and COVID-19 triple co-infection: out of the frying pan into the fire. Cureus.

[5] Haider Z, Ahmad FZ, Mahmood A (2015). Dengue fever in Pakistan: a paradigm shift; changing epidemiology and clinical patterns. Perspect Public Health.

[6] Almas A, Parkash O, Akhter J (2010). Clinical factors associated with mortality in dengue infection at a tertiary care center. Southeast Asian J Trop Med Public Health.

[7] Gupta M, Agrawal N, Sharma SK, Ansari AK, Mahmood T, Singh L (2022). Study of utility of basic arterial blood gas parameters and lactate as prognostic markers in patients with severe dengue. Cureus.

[8] Uthraraj NS, Sriraam LM, Hiriyur Prakash M, Kumar M, Palanisamy U, Chettiakkapalayam Venkatachalam KU (2022). Predictive factors for the complications of dengue fever in children: a retrospective analysis. Cureus.

[9] Roy SK, Bhattacharjee S (2021). Dengue virus: epidemiology, biology, and disease aetiology. Can J Microbiol.

[10] Mallhi TH, Khan AH, Sarriff A, Adnan AS, Khan YH (2017). Determinants of mortality and prolonged hospital stay among dengue patients attending tertiary care hospital: a cross-sectional retrospective analysis. BMJ Open.

[11] Bhaskar ME, Moorthy S, Kumar NS, Arthur P (2010). Dengue haemorrhagic fever among adults - an observational study in Chennai, south India. Indian J Med Res.

[12] Padyana M, Karanth S, Vaidya S, Gopaldas JA (2019). Clinical profile and outcome of dengue fever in multidisciplinary intensive care unit of a tertiary level hospital in India. Indian J Crit Care Med.

[13] Karunakaran A, Ilyas WM, Sheen SF, Jose NK, Nujum ZT (2014). Risk factors of mortality among dengue patients admitted to a tertiary care setting in Kerala, India. J Infect Public Health.

[14] Jain S, Mittal A, Sharma SK (2017). Predictors of dengue-related mortality and disease severity in a tertiary care center in North India. Open Forum Infect Dis.

[15] Khalil MA, Tan J, Khalil MA, Awan S, Rangasami M (2014). Predictors of hospital stay and mortality in dengue virus infection-experience from Aga Khan University Hospital Pakistan. BMC Res Notes.

[16] Ho TS, Wang SM, Lin YS, Liu CC (2013). Clinical and laboratory predictive markers for acute dengue infection. J Biomed Sci.

[17] Thein TL, Leo YS, Fisher DA (2013). Risk factors for fatality among confirmed adult dengue inpatients in Singapore: a matched case-control study. PLoS One.

[18] Medagama A, Dalugama C, Meiyalakan G, Lakmali D (2020). Risk factors associated with fatal dengue hemorrhagic fever in adults: a case control study. Can J Infect Dis Med Microbiol.

[19] Huang HS, Hsu CC, Ye JC, Su SB, Huang CC, Lin HJ (2017). Predicting the mortality in geriatric patients with dengue fever. Medicine (Baltimore).

